# Corrigendum to Effects of switching from liraglutide to semaglutide or dulaglutide in patients with type 2 diabetes: A randomized controlled trial

**DOI:** 10.1111/jdi.14080

**Published:** 2023-09-28

Iijima T, Shibuya M, Ito Y, Terauchi Y. Effects of switching from liraglutide to semaglutide or dulaglutide in patients with type 2 diabetes: A randomized controlled trial. *J Diabetes Investig* 2023; 14: 774–781.

The authors regret inadequacies in the use of the Diabetes Treatment Satisfaction Questionnaire (DTSQ) and its analysis in the original article.

Correction #1

We used a version of the Japanese DTSQ published in 2000 (Ref. 9 in the original article), which is not the most recent authorized version. Problems would not have arisen if we had obtained an authorized Japanese version of the questionnaire and scoring guidelines from the copyright holder; the DTSQ license is available free of charge for non‐commercial research through the site (https://healthpsychologyresearch.com/).

Correction #2

Changes in DTSQ scores, Table 3

We would like to add the following sentence to the original text and also to the legend of Table 3, because the definition of treatment satisfaction on the DTSQ was missing.

Scores on the six DTSQ items (1. Current Treatment, 4. Convenience, 5. Flexibility, 6. Understanding, 7. Recommend, and 8. Continue) were summed to produce a Treatment Satisfaction score.

Correction #3

Changes in DTSQ scores, Table 3

There is no justification for summing items 2 “frequency of hyperglycaemia” and 3 “frequency of hypoglycaemia” on the DTSQ; these items should be analyzed individually^1^, ^2^. The combined score for ‘frequency of hyper‐ and hypoglycaemia should be deleted from Table 3 (which is shown corrected below). The following sentences should be deleted from the original text.

“Additionally, the frequency of hyperglycemia and hypoglycemia in patients in both groups decreased, but not significantly. There was no significant difference between the two groups (*P* = 0.9250).”

**Table 3 revised:** Diabetes Treatment Satisfaction Questionnaire (DTSQ) scores

	Total			Semaglutide (*n* = 16)			Dulaglutide (*n* = 15)			
	Baseline	Week 26	P1	Baseline	Week 26	P2	Baseline	Week 26	P3	P4
No. 1 Current treatment	4.3 ± 1.6	4.9 ± 1.0	0.0172	4.1 ± 1.5	4.7 ± 0.6	0.1643	4.6 ± 1.8	5.2 ± 1.3	0.0572	1.0000
No. 2 Frequency of hyperglycemia	2.4 ± 1.4	1.7 ± 1.5	0.0254	2.8 ± 1.3	1.9 ± 1.2	0.0170	2.0 ± 1.4	1.6 ± 1.7	0.3478	0.5126
No. 3 Frequency of hypoglycemia	0.9 ± 1.4	1.0 ± 1.3	0.9053	0.9 ± 1.4	1.1 ± 1.4	0.6833	0.9 ± 1.4	0.8 ± 1.3	0.6534	0.5974
No. 4 Convenience	3.5 ± 1.6	4.7 ± 1.4	0.0050	3.4 ± 1.8	4.5 ± 1.4	0.0519	3.9 ± 1.6	4.9 ± 1.6	0.0642	0.7045
No. 5 Flexibility	3.3 ± 1.5	4.4 ± 1.2	0.0002	3.3 ± 1.4	4.3 ± 1.0	0.0262	3.5 ± 1.7	4.7 ± 1.4	0.0025	0.6396
No. 6 Understanding	4.1 ± 1.4	4.5 ± 1.2	0.0620	3.9 ± 1.4	4.4 ± 0.9	0.1350	4.5 ± 1.4	4.7 ± 1.4	0.5103	0.5830
No. 7 Recommend	3.9 ± 1.3	4.8 ± 1.2	0.0061	3.5 ± 1.3	4.7 ± 0.8	0.0075	4.3 ± 1.3	4.9 ± 1.5	0.1989	0.3909
No. 8 Continue	3.9 ± 1.7	4.6 ± 1.5	0.0367	3.7 ± 1.5	4.1 ± 1.5	0.3443	4.4 ± 1.8	5.1 ± 1.4	0.1165	0.8436
Treatment Satisfaction	23.1 ± 7.1	28.1 ± 6.7	0.0003	21.8 ± 7.1	26.7 ± 4.8	0.0247	25.1 ± 7.0	29.5 ± 8.0	0.0163	0.6889

P1: Comparison of changes between baseline and week 26 in all cases.

P2: Comparison of changes between baseline and week 26 in the semaglutide group.

P3: Comparison of changes between baseline and week 26 in the dulaglutide group.

P4: Comparison of each level between semaglutide group and dulaglutide group at 26 weeks.

Scores on the six DTSQ scales (1. Current Treatment, 4. Convenience, 5. Flexibility, 6. Understanding, 7. Recommend, and 8. Continue) were summed to provide a Treatment Satisfaction score.

The authors would like to apologize for any inconvenience caused.
